# The flexible linker of the secreted FliK ruler is required for export switching of the flagellar protein export apparatus

**DOI:** 10.1038/s41598-020-57782-5

**Published:** 2020-01-21

**Authors:** Miki Kinoshita, Seina Tanaka, Yumi Inoue, Keiichi Namba, Shin-Ichi Aizawa, Tohru Minamino

**Affiliations:** 1 Graduate School of Frontier Biosciences, Osaka University, 1-3 Yamadaoka, Suita, Osaka 565-0871 Japan; 2 Department of Life Sciences, Prefectural University of Hiroshima, 562 Nanatsuka, Shobara, Hiroshima 727-0023 Japan; 3 RIKEN Spring-8 Center and Center for Biosystems Dynamics Research, 1-3 Yamadoaka, Suita, Osaka 565-0871 Japan; 4 JEOL YOKOGUSHI Research Alliance Laboratories, Osaka University, 1-3 Yamadoaka, Suita, Osaka 565-0871 Japan; 5 Present Address: Department of Ophthalmology and Visual Sciences, Kyoto University Graduate School of Medicine, Kyoto, 606-8507 Japan

**Keywords:** Motor protein function, Bacterial secretion

## Abstract

The hook length of the flagellum is controlled to about 55 nm in *Salmonella*. The flagellar type III protein export apparatus secretes FliK to determine hook length during hook assembly and changes its substrate specificity from the hook protein to the filament protein when the hook length has reached about 55 nm. *Salmonella* FliK consists of an N-terminal domain (FliK_N_, residues 1–207), a C-terminal domain (FliK_C_, residues 268–405) and a flexible linker (FliK_L_, residues 208–267) connecting these two domains. FliK_N_ is a ruler to measure hook length. FliK_C_ binds to a transmembrane export gate protein FlhB to undergo the export switching. FliK_L_ not only acts as part of the ruler but also contributes to this switching event, but it remains unknown how. Here we report that FliK_L_ is required for efficient interaction of FliK_C_ with FlhB. Deletions in FliK_L_ not only shortened hook length according to the size of deletions but also caused a loose length control. Deletion of residues 206–265 significantly reduced the binding affinity of FliK_C_ for FlhB, thereby producing much longer hooks. We propose that an appropriate length of FliK_L_ is required for efficient interaction of FliK_C_ with FlhB.

## Introduction

The bacterial flagellar hook is a tubular structure composed of the hook protein FlgE and acts as a universal joint to smoothly transmit torque produced by the flagellar motor to the long helical filament that functions as a propeller^1^. The hook length of the *Salmonella* flagellum is controlled to about 55 nm^2^, and the length control is important for the universal joint mechanism^3^.

The flagellar type III protein export apparatus is located at the flagellar base and transports FlgE subunits from the cytoplasm to the distal end of the growing hook structure for hook assembly^4,5^. Newly exported FlgE monomers polymerize onto the nascent hook structure with the help of the hook cap (FlgD), which is located at the hook tip^6^. Interactions between FlgD and FlgE suppress the leakage of FlgE subunits into the culture media so that each FlgE subunit can be efficiently incorporated into the hook structure^7,8^.

When hook length has reached about 55 nm, the flagellar type III protein export apparatus changes its substrate specificity, thereby terminating hook assembly and initiating filament assembly^9,10^. FliK, FlhA, FlhB and Flk are directly involved in the export switching process^11–14^. If certain mutations in FliK, FlhB or FlhA inhibits the export switching of the flagellar type III protein export apparatus, unusually elongated hooks called polyhooks are generated. FlhA and FlhB are transmembrane proteins of the flagellar protein export apparatus^15,16^, and their C-terminal cytoplasmic domains (FlhA_C_, FlhB_C_) project into the cavity within the basal body C ring^15,16^. FlhA_C_ forms a homo-nonameric ring structure^17,18^ and provides binding-sites for cytoplasmic export components (FliH, FliI, FliJ)^19–21^ and flagellar export chaperones (FlgN, FliS, FliT) in with their cognate substrates^22–25^. Interactions of FlhA_C_ with FliH, FliI and FliJ seems to support the strict order of flagellar assembly^26,27^. The interaction of FlhB_C_ with FliK induces the export switching of the flagellar type III protein export apparatus^28–30^. FlhB_C_ binds to FliH, FliI, FliJ and export substrates such as FlgD and FlgE^19,31,32^. Conformational changes of FlhA_C_ and FlhB_C_ are required for the substrate specificity switching of the flagellar protein export apparatus upon hook completion^18,28,33^. Flk interferes with premature switching of the protein export apparatus during hook-basal body assembly^13,14,34^.

**Figure 1:**
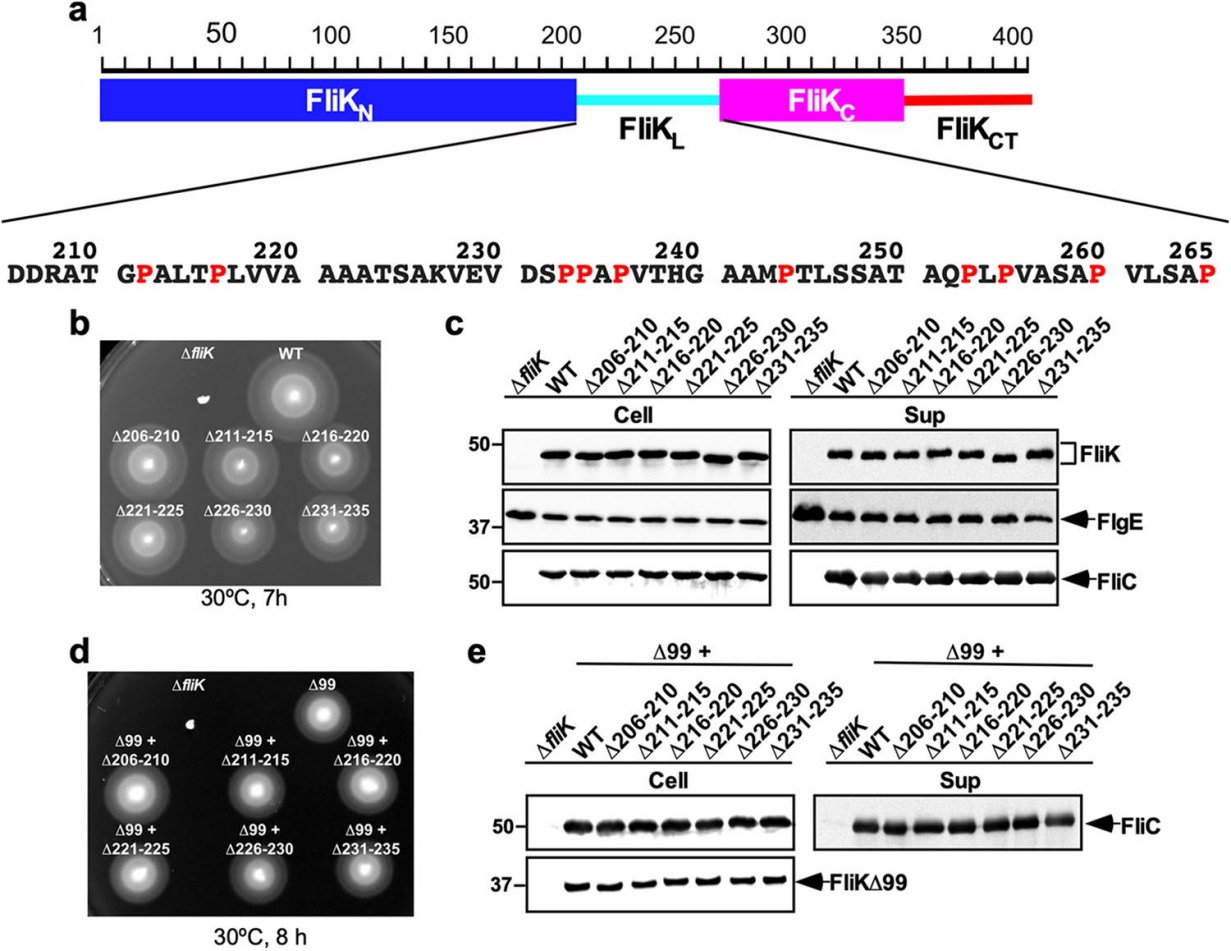
Effect of deletions of five residues within the N-terminal region of FliK_L_ on FliK function. (**a**) Domain organization of FliK ruler. FliK consists of the N-terminal ruler domain (FliK_N_), the C-terminal export switch domain (FliK_C_) and a flexible linker (FliK_L_) connecting these two domains. FliK_C_ has an intrinsically disordered C-terminal tail (FliK_CT_). Amino acid sequence of FliK_L_ is shown. Proline residues are highlighted in red. (**b**) Motility of TH8426 harboring pTrc99AFF4 (∆*fliK*), pMK002 (WT), pMMK1001 (∆206–210), pMMK1002 (∆211–215), pMMK1003 (∆216–220), pMMK1004 (∆221–225), pMMK1005 (∆226–230) or pMMK1006 (∆231–235) in 0.35% soft agar. Plates were incubated at 30 °C for 7 hours. (**c**) Secretion assays of FliK, FlgE and FliC. Immunoblotting using polyclonal anti-FliK (1st row), anti-FlgE (2nd row) or anti-FliC (3rd row) antibody, of whole cell proteins (Cell) and culture supernatants (Sup) from the above strains. The regions of interest were cropped from original immunoblots shown in Fig. S6a in the Supplemental information using a software, Photoshop CS6, and then the contrast and brightness were adjusted. The positions of molecular mass markers (kDa) are indicated on the left. (**d**) Motility of TH8426 harboring pTrc99AFF4 (∆*fliK*), pNM201 (∆99), pMMK1016 (∆99 + ∆206–210), pMMK1017 (∆99 + ∆211–215), pMMK1018 (∆99 + ∆216–220), pMMK1019 (∆99 + ∆221–225), pMMK1020 (∆99 + ∆226–230) or pMMK1021 (∆99 + ∆231–235) in 0.35% soft agar containing 1 mM IPTG. Plates were incubated at 30 °C for 8 hours. (**e**) Secretion assay of FliC. Immunoblotting using polyclonal anti-FliC (1st row) or anti-FliK (2nd row) antibody, of whole cell proteins (Cell) and culture supernatants (Sup) from the above strains. The regions of interest were cropped from original immunoblots shown in Fig. S6b using Photoshop CS6, and then the contrast and brightness were adjusted.

The flagellar type III protein export apparatus transports several FliK molecules during hook assembly to determine hook length in a way that the protein export apparatus switches its substrate specificity when the hook length has reached about 55 nm^35–38^. *Salmonella* FliK is a 405 amino-acid protein consisting of the N-terminal ruler domain (FliK_N_, residues 1–207), the C-terminal export switch domain (FliK_C_, residues 268–405) and a flexible linker (FliK_L_, residues 208–267) connecting these two domains (Fig. 1a)^39–41^. FliK_N_ contains a hook-type export signal recognized by the flagellar type III protein export apparatus^31,35^. Insertions and deletions in FliK_N_ make the hook longer and shorter, respectively, suggesting that FliK_N_ is a molecular ruler^42^. FliK_N_ binds to FlgD and FlgE^43–45^, and deletion of residues 129–159 in FliK_N_ reduces the binding affinity of FliK for FlgE, thereby producing longer hooks with the filament attached. This suggests that the interaction of FliK_N_ with FlgE is required for proper hook length measurement^44^. FliK_C_ consists of a compactly folded core domain consisting of residues 268–352 and an intrinsically disordered C-terminal tail composed of residues 353–405 (FliK_CT_) (Fig. 1a)^40,46^. The core domain of FliK_C_ binds to FlhB_C_ to allow the flagellar protein export apparatus to switch its substrate specificity from rod- (FlgB, FlgC, FlgF, FlgG, FlgJ, FliE) and hook-type (FlgD, FlgE, FliK) to filament-type (FlgK, FlgL, FlgM, FliC, FliD)^11,12,28,45,47^. Genetic analyses of FliK_CT_ have suggested that FliK_CT_ controls the export switching activity of FliK_C_ during hook assembly^45,47^.

FliK_L_ contains ten proline residues (Fig. 1a)^39^ and hence is intrinsically disordered^41^. Hook lengths of the *Salmonella fliK*(**∆**238–269) (32 residues deletion) and *fliK*(**∆**248–269) (22 residues deletion) mutants are 40.2 ± 6.1 nm [mean ± standard deviation (SD)] and 51.0 ± 8.8 nm, where their average lengths are shorter than that of the wild-type strain (52.7 ± 4.5 nm)^42^. The length of the hook produced by the *fliK*(**∆**161–216) mutant (56 residues deletion) is 48.7 ± 22.3 nm, where the average is also shorter than that of the wild-type strain by 4 nm. However, the SD value is larger than the wild-type one, indicating a much looser length control of the hook structure^42^. Furthermore, deletions of residues 161–223 and 161–244 cause polyhooks without the filament attached whereas a deletion of residues 208–269 results in the polyhooks with the filament attached (polyhook–filament phenotype)^42^. These observations raise the possibility that FliK_L_ not only acts as part of the ruler but also contributes to substrate specificity switching of the flagellar protein export apparatus. To clarify this hypothesis, we constructed a series of mutant variants of FliK with in-frame deletions within FliK_L_. We show that a proper length of FliK_L_ between FliK_N_ and FliK_C_ is required for efficient interaction of FliK_C_ with FlhB_C_.

## Results

### Effect of deletions of five amino-acid residues within residues 206–235 on hook length control

It has been shown that the N-terminal portion of FliK_L_ is responsible for proper measurement of hook length^42^. To clarify the role of residues 206–235 of FliK_L_ in the hook length control, we constructed a series of mutant variants of FliK with sequential 5-amino-acid deletions within a region of residues 206–235, namely FliK(∆206–210), FliK(∆211–215), FliK(∆216–220), (∆221–225), (∆226–230) and FliK(∆231–235) (Table 1). These six *fliK* deletion variants fully restored motility of the ∆*fliK* mutant in 0.35% soft agar plates when they were expressed from the pTrc99A-based plasmid (Fig. 1b). Consistently, the levels of FlgE and FliC secreted by these deletion mutants were detected at the wild-type levels (Fig. 1c, 2nd and 3rd rows). These *fliK* deletions did not affect either protein stability or protein secretion into the culture media (Fig. 1c, 1st row). Therefore, we conclude that these in-frame deletions do not affect FliK function at all.

**Table 1:** Strains and plasmids used in this study.

Strains and Plasmids	Relevant characteristics	Source or reference
*E. coli*		
BL21 Star (DE3)	Overexpression of proteins	Novagen
*Salmonella*		
SJW1103	Wild type for motility and chemotaxis	^61^
TH8426	∆*fliK*	^47^
MMK1012	*fliK*(∆206–235)	This study
MMK1013	*fliK*(∆206–245)	This study
MMK1014	*fliK*(∆206–255)	This study
MMK1015	*fliK*(∆206–265)	This study
Plasmids		
pTrc99AFF4	Modified pTrc expression vector	^62^
pKM002	pTrc99AFF4/FliK	^48^
pNM201	pTrc99AFF4/FliK(∆2–99)	^44^
pMMK521	pETDuet-1/FliK(I304amber) + FlhB_C_	^45^
pMMK1001	pTrc99AFF4/FliK(∆206–210)	This study
pMMK1002	pTrc99AFF4/FliK(∆211–215)	This study
pMMK1003	pTrc99AFF4/FliK(∆216–220)	This study
pMMK1004	pTrc99AFF4/FliK(∆221–225)	This study
pMMK1005	pTrc99AFF4/FliK(∆226–230)	This study
pMMK1006	pTrc99AFF4/FliK(∆231–235)	This study
pMMK1007	pTrc99AFF4/FliK(∆206–215)	This study
pMMK1008	pTrc99AFF4/FliK(∆216–225)	This study
pMMK1009	pTrc99AFF4/FliK(∆226–235)	This study
pMMK1010	pTrc99AFF4/FliK(∆206–220)	This study
pMMK1011	pTrc99AFF4/FliK(∆221–235)	This study
pMMK1012	pTrc99AFF4/FliK(∆206–235)	This study
pMMK1013	pTrc99AFF4/FliK(∆206–245)	This study
pMMK1014	pTrc99AFF4/FliK(∆206–255)	This study
pMMK1015	pTrc99AFF4/FliK(∆206–265)	This study
pMMK1015SP	pTrc99AFF4/FliK(∆206–265SP)	This study
pMMK1016	pTrc99AFF4/FliK(∆2–99 + ∆206–210)	This study
pMMK1017	pTrc99AFF4/FliK(∆2–99 + ∆211–215)	This study
pMMK1018	pTrc99AFF4/FliK(∆2–99 + ∆216–220)	This study
pMMK1019	pTrc99AFF4/FliK(∆2–99 + ∆221–225)	This study
pMMK1020	pTrc99AFF4/FliK(∆2–99 + ∆226–230)	This study
pMMK1021	pTrc99AFF4/FliK(∆2–99 + ∆231–235)	This study
pMMK1022	pTrc99AFF4/FliK(∆2–99 + ∆206–215)	This study
pMMK1023	pTrc99AFF4/FliK(∆2–99 + ∆216–225)	This study
pMMK1024	pTrc99AFF4/FliK(∆2–99 + ∆226–235)	This study
pMMK1025	pTrc99AFF4/FliK(∆2–99 + ∆206–220)	This study
pMMK1026	pTrc99AFF4/FliK(∆2–99 + ∆221–235)	This study
pMMK1027	pTrc99AFF4/FliK(∆2–99 + ∆206–235)	This study
pMMK1028	pTrc99AFF4/FliK(∆2–99 + ∆206–245)	This study
pMMK1029	pTrc99AFF4/FliK(∆2–99 + ∆206–255)	This study
pMMK1030	pTrc99AFF4/FliK(∆2–99 + ∆206–265)	This study
pMMK1030SP	pTrc99AFF4/FliK(∆2–99 + ∆206–265SP)	This study
pMMK1031	pETDuet-1/FliK(∆2–99 + I304amber) + FlhB_C_	This study
pMMK1032	pETDuet-1/FliK(∆2–99 + ∆206–265 + I304amber) + FlhB_C_	This study
pMMK1032SP	pETDuet-1//FliK(∆2–99 + ∆206–265SP + I304amber) + FlhB_C_	This study

The length of the most extended polypeptide chain is 0.37 nm per residue. If FliK_L_ adopts a fully extended conformation to act as part of the ruler, we predicted that these 5-amino-acid deletions within residues 206–235 of FliK_L_ would reduce the hook length by 1.9 nm. Therefore, we measured the hook length of these *fliK* deletion mutants. The average hook length of the *fliK*(∆206–210), *fliK*(∆211–215), *fliK*(∆216–220), *fliK*(∆221–225), *fliK*(∆226–230) and *fliK*(∆231–235) mutants were 48.5 ± 4.5 nm (n = 113), 49.3 ± 5.2 nm (n = 168), 49.0 ± 5.6 nm (n = 130), 48.6 ± 4.2 nm (n = 118), 49.2 ± 4.2 nm (n = 232) and 49.6 ± 5.4 nm (n = 126), respectively, which were shorter than the length of the wild-type hook (53.3 ± 6.5 nm, n = 154) (Fig. S1). Over-expression of FliK slightly shortens the hook length. In contrast, when the expression level of FliK is reduced, the cell produces polyhooks, sometimes with the filament attached^27,48^. Polyhooks are frequently observed when FlgE is overproduced in wild-type cells^27,43^. Thus, the balance between the secretion levels of FlgE and FliK seems to be critical for the proper termination of the hook assembly. Since 5-amino-acid deletions within residues 206–235 shorten the hook length by 4 nm, which is shorter than the predicted value, we assume that these deletions may affect not only hook length measurements but also the secretion process of FliK by the type III protein export apparatus and/or the export switching process of the protein export apparatus induced by the interaction of FliK_C_ with FlhB_C_.

To directly test if these 5 amino-acid deletions affect the interaction of FliK_C_ with FlhB_C_, it is necessary to analyze the protein transport process of FliK and the export switching process of the flagellar type III protein export apparatus separately. To do so, we used a *fliK*(**∆**2–99) allele, which is an export deficient variant of FliK, because it retains the ability to catalyze export switching of the flagellar type III protein export apparatus when over-expressed^49^. We introduced deletions of residues 206–210, 211–215, 216–220, 221–225, 226–230 or 231–235 into the *fliK*(**∆**2–99) allele by inverse PCR method and analyzed motility of the ∆*fliK* mutant cells over-expressing FliK(∆2–99) with these deletions in the presence of 1 mM isopropyl β-D-1-thiogalactopyranoside (IPTG). These additional deletions did not affect motility of the cells over-expressing FliK(∆2–99) (Fig. 1d). Consistently, the inner and extracellular amounts of FliC seen in the *fliK*(∆2–99 + ∆206–210), *fliK*(∆2–99 + ∆211–215), *fliK*(∆2–99 + ∆216–220), *fliK*(∆2–99 + ∆221–225), *fliK*(∆2–99 + ∆226–230) and *fliK*(∆2–99 + ∆231–235) cells were the same as those in the *fliK*(∆2–99) cells (Fig. 1e, 1st row). Since these deletions did not affect the expression level of FliK(∆2–99) (Fig. 1e, 2nd row), we suggest that they do not affect the interaction of FliK(∆2–99) with FlhB_C_. Therefore, we propose that these deletions may affect the protein transport process of FliK as well as hook length measurement.

**Figure 2:**
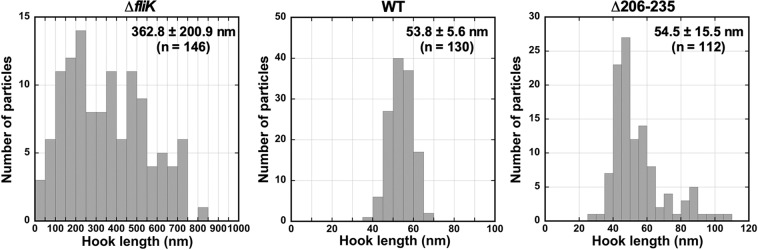
Effect of deletion of residues 206–235 of FliK_L_ on hook length control. Histograms of hook length distribution of TH8426 (∆*fliK*), SJW1103 (WT) and MMK1012 (∆206–235).

To further understand the ruler function of residues 206–235 in FliK_L_, we constructed larger deletion variants, FliK(∆206–215), FliK(∆216–225), FliK(∆226–235), FliK(∆206–220), FliK(∆221–235) and FliK(∆206–235) and analyzed their motility in 0.35% soft agar plates (Fig. S2a). Motility of the *fliK*(∆206–215), *fliK*(∆226–235), *fliK*(∆206–220), *fliK*(∆221–235) and *fliK*(∆206–235) cells was almost the same as wild-type motility whereas that of the *fliK*(∆216–225) mutant was slightly less than the wild-type level (Fig. S2a). The cellular and secretion levels of these deletion variants were essentially the same as the wild-type levels (Fig. S2b). Because these deletion mutants were expressed from the pTrc99AFF4 vector, it is also possible that their over-expression results in motility comparable to the wild-type level. To verify this possibility, the wild-type *fliK* allele on the chromosomal DNA was replaced by the *fliK*(∆206–235) allele. Motility of the *fliK*(∆206–235) mutant was slightly less than that of wild-type cells (Fig. S2c). To test whether the deletion of residues 206–235 affect hook length, we isolated hook-basal bodies from the *fliK*(∆206–235) mutant and measured the hook length (Figs. 2 and S3). A major peak of the hook length distribution was shifted to a shorter value than that of the wild-type (Fig. 2). While the majority of wild-type hook length was distributed within a range from 50 nm to 60 nm, the hook length distribution of the *fliK*(∆206–235) mutant showed a major peak population between 41 nm and 50 nm. However, longer hooks were also observed albeit shorter than those of polyhooks produced by the ∆*fliK* mutant [362.8 ± 200.9 nm (N = 146)]. As a result, the average hook length of the *fliK*(∆206–235) mutant was 54.5 ± 15.5 nm (n = 112), compared to 53.8 ± 5.6 nm (n = 130) for the wild-type. The SD value of the *fliK*(∆206–235) mutant was larger than the wild-type one, indicating that the deletion of residues 206–235 in FliK_L_ cause a looser hook length control than the wild-type. Therefore, we suggest that a deletion of residues 206–235 not only shortens the hook length according to the size of deletion but also affects the interaction of FliK_C_ with FlhB_C_ during hook assembly.

### Effect of much larger deletions within FliK_L_ on hook length control and substrate specificity switching

**Figure 3:**
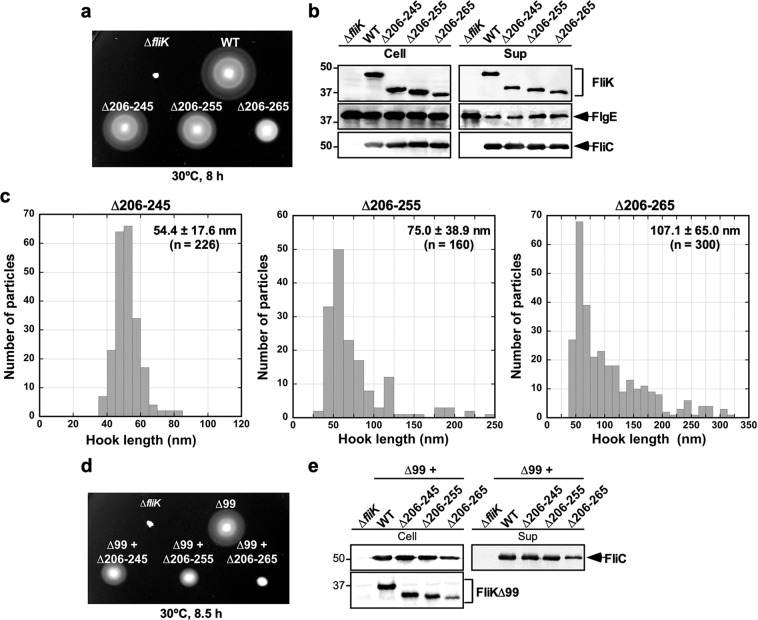
Effect of deletion of residues 206–245, 206–255 or 206–265 on FliK function. (**a**) Motility of TH8426 (∆*fliK*), SJW1103 (WT), MMK1013 (∆206–245), MMK1014 (∆206–255) and MMK1015 (∆206–265) in 0.35% soft agar. Plates were incubated at 30 °C for 8 hours. (**b**) Secretion assays of FliK, FlgE and FliC. Immunoblotting using polyclonal anti-FliK (1st row), anti-FlgE (2nd row) or anti-FliC (3rd row) antibody, of whole cell proteins (Cell) and culture supernatants (Sup) from the above strains. The regions of interest were cropped from original immunoblots shown in Fig. S7a in the Supplemental information using Photoshop CS6, and then the contrast and brightness were adjusted. The positions of molecular mass markers (kDa) are indicated on the left. (**c**) Histograms of hook length distribution of MMK1013 (∆206–245), MMK1014 (∆206–255) and MMK1015 (∆206–265). (d) Motility of TH8426 harboring pTrc99AFF4 (∆*fliK*), pNM201 (∆99), pMMK1028 (∆99 + ∆206–245), pMMK1029 (∆99 + ∆206–255) or pMMK1030 (∆99 + ∆206–265) in 0.35% soft agar containing 1 mM IPTG. Plates were incubated at 30 °C for 8 hours. (**e**) Secretion assay of FliC. Immunoblotting using polyclonal anti-FliC (1st row) or anti-FliK (2nd row) antibody, of whole cell proteins (Cell) and culture supernatants (Sup) from the above strains. The regions of interest were cropped from original immunoblots shown in Fig. S7b in the Supplemental information using Photoshop CS6, and then the contrast and brightness were adjusted. The positions of molecular mass markers (kDa) are indicated on the left.

To clarify the role of FliK_L_ in the export switching process of the flagellar type III protein export apparatus, the wild-type *fliK* allele on the chromosome was replaced by the *fliK*(∆206–245), *fliK*(∆206–255) or *fliK*(∆206–265) allele. Motility of these mutants were worse than wild-type motility (Fig. 3a) although the cellular and secreted amounts of FlgE and FliC were detected almost at the wild-type levels (Fig. 3b, 2nd and 3rd rows). Because neither cellular nor extracellular FliK level was affected by these larger deletions within FliK_L_ (Fig. 3b, 1st row), this suggests that these deletions reduce FliK function.

To test whether these larger deletions affect the hook length control, we measured the hook length of the *fliK*(∆206–245), *fliK*(∆206–255) and *fliK*(∆206–265) mutants (Figs. 3c and S3). The hook length distribution of the *fliK*(∆206–245) mutant shows a major peak population between 45 nm and 55 nm. Longer hooks and polyhooks were seen for the *fliK*(∆206–255) and *fliK*(∆206–265) mutants although shorter hooks were also observed (Fig. S3). As a result, the average hook lengths of the *fliK*(∆206–245), *fliK*(∆206–255) and *fliK*(∆206–265) mutants were 54.4 ± 17.6 nm (n = 226), 75.0 ± 38.9 nm (n = 160) and 107.1 ± 65.0 nm (n = 300), respectively, indicating that the hook length control becomes worse in the *fliK*(∆206–255) and *fliK*(∆206–265) mutants.

To investigate whether these larger deletions directly affect the export switching function of FliK_C_, we introduces deletions residues 206–245, 206–255 or 206–265 into the *fliK*(∆2–99) allele. Motility of the cells over-expressing FliK(∆2–99) with these three deletions was reduced significantly (Fig. 3d), and especially the deletion of residues 206–265 reduced the cellular and extracellular levels of FliC by about 3-fold, thereby reducing motility considerably (Fig. 3e). This suggests that these three deletions reduce the export switching function of FliK_C_.

**Figure 4:**
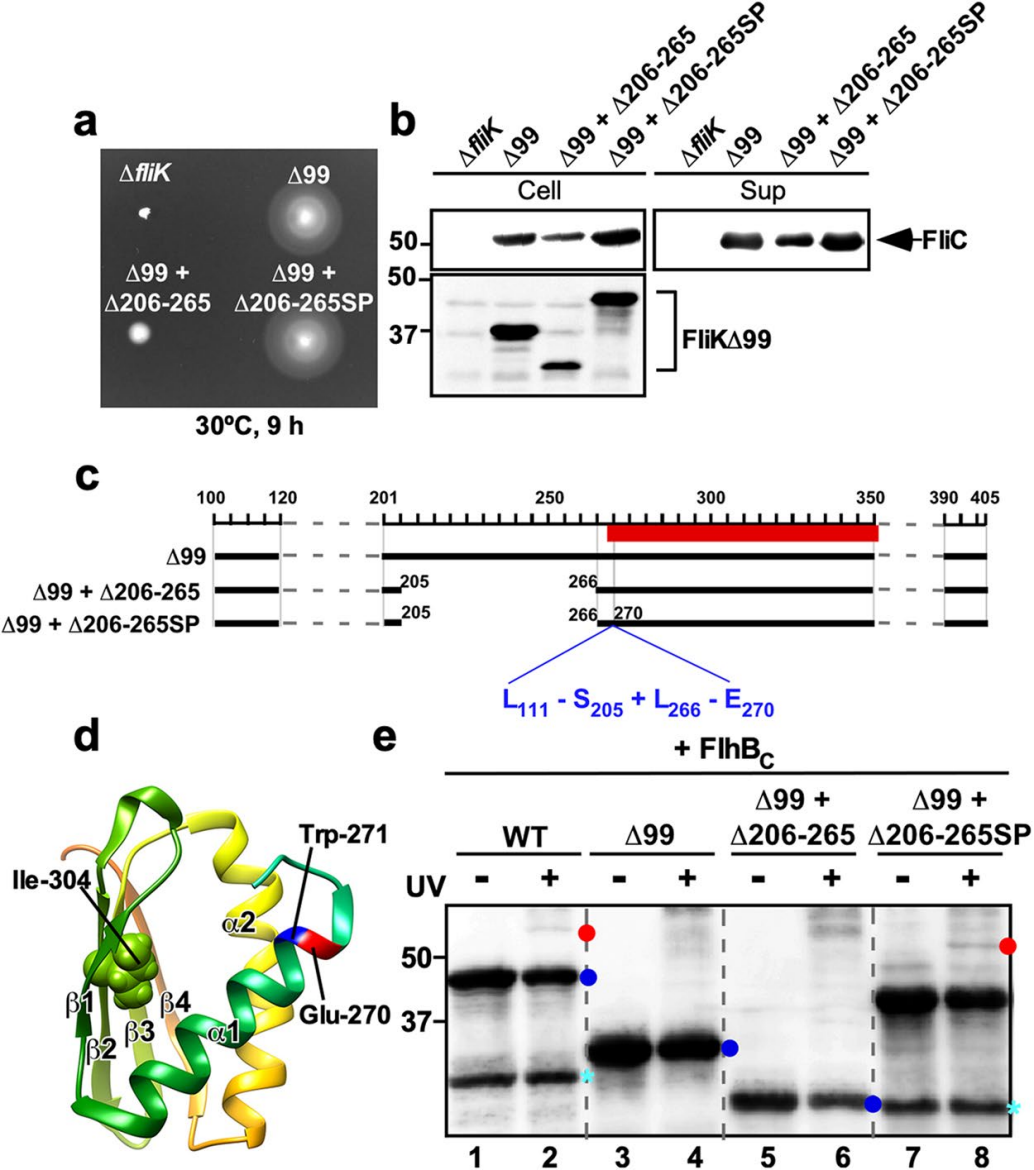
Isolation of pseudorevertants from the *fliK*(∆99 + ∆206–265) mutant. (**a**) Motility of TH8426 transformed with pTrc99A (∆*fliK*), pNM201 (∆99), pMMK1030 (∆99 + ∆206–265) or pMMK1030SP (∆99 + ∆206–265SP) in 0.35% soft agar containing 1 mM IPTG. Plates were incubated at 30 °C for 9 hours. (**b**) Secretion assay of FliC. Immunoblotting using polyclonal anti-FliC (1st row) or anti-FliK (2nd row) antibody, of whole cell proteins (Cell) and culture supernatants (Sup) prepared from the above strains. The regions of interest were cropped from original immunoblots shown in Fig. S8a in the Supplemental information using Photoshop CS6, and then the contrast and brightness were adjusted. The positions of molecular mass markers (kDa) are indicated on the left. (**c**) Location of the intragenic suppressor insertion mutation on FliK(∆99 + ∆206–265) and schematic bar diagram representations of FliK∆99 products caused by a deletion of residues 206–265 and the intragenic suppressor insertion of residues 111–270 lacking residues 206–265 (L_111_– S_205_ + L_266_–E _270_) between Glu-270 and Trp-271 residues. Red bar indicates a core domain of FliK_C_. (**d**) NMR structure of a core domain of FliK_C_ (PDB ID: 2RRL). The Cα backbone is color-coded from green to orange, going through the rainbow colors from the N- to the C-terminus. A highly conserved Ile-304 residue is involved in the interaction with FlhB. The inserted suppressor sequence is located between Glu-270 and Trp-271 residues. (**e**) Photo-crosslinking between FliK(∆2–99) and FlhB_C_. *E. coli* BL21(DE3) cells co-expressing FliK(I304pBPA), FliK(∆99 + I304pBPA), FliK(∆99 + ∆206–265 + I304pBPA) or FliK(∆99 + ∆206–265SP + I304pBPA) with FhB_C_ were UV-irradiated for 5 min (+) or not irradiated (−), and then analyzed by immunoblotting with polyclonal anti-FliK antibody. The regions of interest were cropped from original immunoblots shown in Fig. S8b in the Supplemental information using Photoshop CS6, and then the contrast and brightness were adjusted. The positions of molecular mass markers (kDa) are indicated on the left. The positions of free FliK and FliK-FlhB_C_ photo-crosslinked products are shown by blue and red balls, respectively. C-terminal truncated variants of FliK are shown by cyan asterisk.

**Figure 5:**
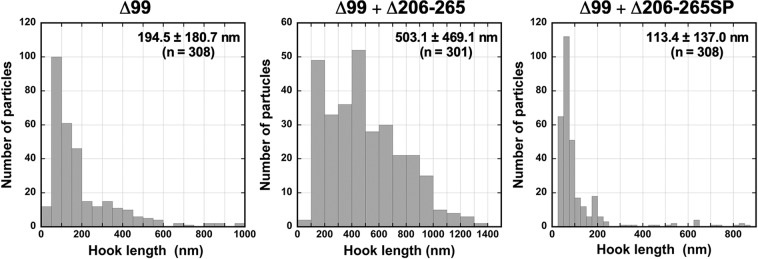
Effects of deletion of residues 206–265 and its intragenic suppressor insertion mutation on length distribution of the hooks produced by the *fliK*(∆2–99) mutant. Histograms of hook length distribution of TH8426 harboring pNM201 (∆99), pMMK1030 (∆99 + ∆206–265) or pMMK1030SP (∆99 + ∆206–265SP).

To clarify why the deletion of residues 206–265 considerably reduces the export switching activity of FliK_C_, we isolated fourteen pseudorevertants from the cells over-expressing FliK(∆2–99) with an in-frame deletion of residues 206–265. Motility of these pseudorevertants was much better than that of its parent cells and was slightly better than that of the cells over-expressing FliK(**∆**2–99) (Fig. 4a). Consistently, the cellular and extracellular levels of FliC seen in these pseudorevertants were much higher than those in the *fliK*(∆2–99 + ∆206–265) cells and slightly larger than those in the *fliK*(∆2–99) cells (Fig. 4b). These results indicate that the suppressor mutations enhance the probability of export switching to occur. DNA sequence analysis revealed that all suppressor mutations are insertion mutation of a DNA fragment encoding 100 amino-acid residues corresponding to residues 111–270 without residues 206–265 due to gene duplication (Fig. 4c). As a result, this intragenic FliK(∆2–99 + ∆206–265) suppressor mutation variant, namely FliK(∆2–99 + ∆206–265SP), showed much slower mobility on SDS-PAGE than FliK(∆2–99) and FliK(∆2–99 + ∆206–265) (Fig. 4b). The inserted sequence of the suppressor mutant was located between Glu-270 and Trp-271 residues in helix α1 of the core domain of FliK_C_ (Fig. 4d). The *fliK*(∆2–99 + ∆206–265) mutant cells produced polyhooks sometimes with filaments attached (Fig. S4). In contrast, most of the pseudorevertant cells had a few flagella (Fig. S4). The pseudorevertant cells produced normal hooks or much shorter polyhooks compared to the *fliK*(∆2–99) and *fliK*(∆2–99 + ∆206–265) cells (Fig. S4). Careful measurements of the hook length indicated that the average hook length of the pseudorevertant were 113.4 ± 136.7 nm (n = 308), compared to 194.5 ± 180.4 nm (n = 308) for the *fliK*(∆2–99) cells and 503.1 ± 281.3 nm (n = 301) for the *fliK*(∆2–99 + ∆206–265) cells (Figs. 5 and S5). These results led to a conclusion that FliK_L_ is required for efficient substrate specificity switching of the flagellar type III protein export apparatus.

### Interaction between FliK(∆2–99) and FlhB_C_

Targeted photo-crosslinking experiments have shown that Val-302 and Ile-304 of FliK_C_ are in relatively close proximity of FlhB_C_, allowing FliK to form a photo-crosslinked product with FlhB_C_^45^. To investigate whether the deletion of residues 206–265 of FliK_L_ and its suppressor insertion mutation reduces and increases the binding affinity of FliK_C_ for FlhB_C_, respectively, we introduced an amber mutation at position 304 of FliK(∆2–99), FliK(∆2–99 + ∆206–265) and FliK(∆2–99 + ∆206–265SP) to incorporate *p*-benzoyl-phenylalanine (pBPA), which is a photo-reactive phenylalanine and carried out photo-crosslinking experiments. We used FliK(I304amber) as a positive control. FliK(I304pBPA) produced a ca. 53 kDa photo-crosslinked product with FlhB_C_ after UV irradiation (Fig. 4e, lane 2), in agreement with a previous report^45^. However, FliK(∆2–99 + I304pBPA) did not form any photo-crosslinked products with FlhB_C_ (Fig. 4e, lane 4), indicating that the binding affinity of FliK(∆2–99) for FlhB_C_ is lower than that of wild-type FliK. FliK(∆2–99 + ∆206–265SP + I304pBPA) reproducibly formed a 51 kDa photo-crosslinked product along with FlhB_C_ (Fig. 4e, lane 8) whereas FliK(∆2–99 + ∆206–265 + I304pBPA) did not (lane 6). This suggests that the inserted sequence of the pseudorevertant increases the binding affinity of FliK(∆2–99) for FlhB_C_ so that the export switching of the type III protein export apparatus occurs more efficiently.

### Effect of the 100 residues suppressor insertion on the length of hook produced by the *fliK*(∆206–265) mutant

**Figure 6:**
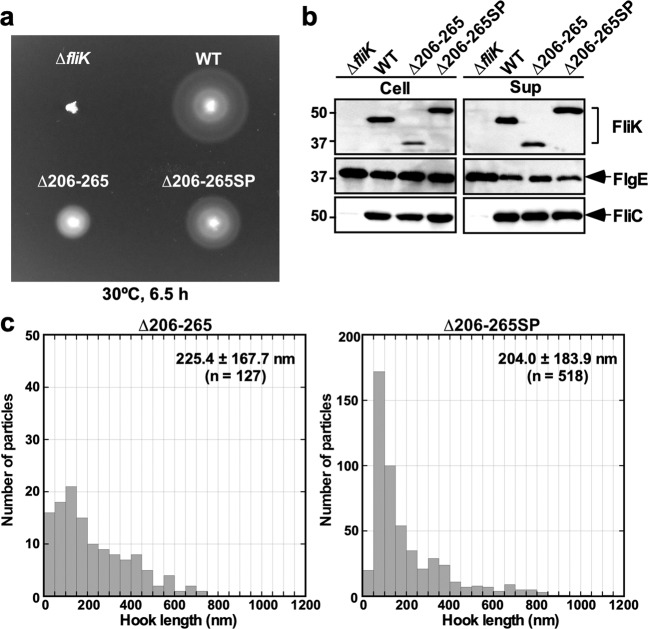
Effects of the inserted sequence of the intragenic *fliK*(∆2–99 + ∆206–265) suppressor mutant on length distribution of the hooks produced by the *fliK*(∆206–265) mutant. (**a**) Motility of TH8426 harboring pTrc99A (∆*fliK*), pKM002 (WT), pMMK1015 (∆206–265) or pMMK1015SP (∆206–265SP) in 0.35% soft agar. Plates were incubated at 30 °C for 6.5 hours. (**b**) Secretion assays of FlgE and FliC. Immunoblotting using polyclonal anti-FliK (1st row), anti-FlgE (2nd row) or anti-FliC (3rd row) antibody, of whole cell proteins (Cell) and culture supernatants (Sup) prepared from the above strains. The regions of interest were cropped from original immunoblots shown in Fig. S9 using Photoshop CS6, and then the contrast and brightness were adjusted. The positions of molecular mass markers (kDa) are indicated on the left. (**c**) Histograms of hook length distribution of TH8426 harboring pMMK1015 or pMMK1015SP.

To investigate whether the insertion mutation of the *fliK*(∆2–99 + ∆206–265) suppressor mutant also improves the export switching function of the *fliK*(∆206–265) mutant, we introduced the extra 100 amino-acid insertion sequence of the *fliK*(∆2–99 + ∆206–265SP) mutant into the *fliK*(∆206–265) allele by the overlap PCR method to generate the *fliK*(∆206–265SP) allele. Motility of the ∆*fliK* mutant harboring pMMK1015SP [FliK(∆206–265SP)] was better than that of the ∆*fliK* mutant harboring pMMK1015 [FliK(∆206–265)] although it was worse than that of the ∆*fliK* mutant transformed with pKM002 (wild-type FliK) (Fig. 6a). Neither cellular nor extracellular FliK level was affected by the inserted sequence (Fig. 6b, 1st row). The amounts of FlgE secreted by the *fliK*(∆206–265SP) mutant were slightly less than that by the *fliK*(∆206–265) mutant and almost the same as the wild-type level (Fig. 6b, 2nd and 3rd rows). There was no difference in the cellular and extracellular FliC levels between the *fliK*(∆206–265) and *fliK*(∆206–265SP) mutants. These results indicates that the inserted sequence of the *fliK*(∆2–99 + ∆206–265) suppressor mutant is also capable of improving the export switching function of FliK(∆206–265).

The amino acid sequence of FliK(∆206–265SP) is longer by 40 amino-acids than that of wild-type FliK, from which the average hook length is predicted to be about 70 nm, thereby reducing motility. To verify this hypothesis, we isolated hook-basal bodies from the ∆*fliK* mutant carrying pMMK1015 [FliK(∆206–265)] or pMMK1015SP [FliK(∆206–265SP)] and measured their hook length (Figs. 6c and S5). The average hook length of the ∆*fliK* mutant carrying pMMK1015 was 225.4 ± 167.7 nm (n = 127), which were longer than that of the MMK1015 strain (107.1 ± 65.0 nm). Since FliK(∆206–265) was expressed from the pTrc99AFF4 vector, we assume that such a length difference may be a consequence of the multicopy effect of FliK(∆206–265SP). The hook length distribution of the ∆*fliK* mutant carrying pMMK1015SP showed a major peak population between 61 nm and 100 nm, but much longer hooks and polyhooks were observed as well. As a result, the average hook length of the ∆*fliK* mutant carrying pMMK1015SP was 204.0 ± 183.9 nm (n = 518), which was shorter than that of the ∆*fliK* mutant carrying pMMK1015. This suggests that the suppressor insertion mutation increases the probability of the interaction between FliK_C_ with a deletion of residue 206–265 and FlhB_C_, thereby increasing the export switching probability of the flagellar type III protein export apparatus.

## Discussion

The bacterial injectisome directly transports virulence effector proteins into the cytosol of host cells for bacterial infection. The injectisome consists of basal body rings and a tubular structure called the needle and looks similar to the flagellar hook-basal body^50^. The injectisome uses a secreted molecular ruler, SctP (originally referred to as YscP and InvJ in the *Yeshinia* and *Salmonella* injectisomes, respectively) to determine the needle length in a way similar to FliK^51,52^. The core domain of FliK_C_ is conserved among FliK/SctP family^53^ and possesses a fold similar to the C-terminal domain of SctP of the injectisome of *Pseudomonas aeruginosa*^54^. It has been shown that residues 301–350 of FliK_C_ are directly involved in substrate specificity switching of the flagellar type III protein export apparatus^47^. Recent photo-crosslinking experiments have demonstrated that the conserved core domain of FliK_C_ directly binds to FlhB_C_^45^. Similar protein-protein interactions have been observed in the injectisome^55^. These suggest that length control and substrate specificity switching mechanisms are conserved in both flagellar and injectisome systems. However, it remains unknown how the length measurement process by the secreted ruler is linked to the substrate specificity switching process of the type III protein export apparatus.

It has been reported that FliK_L_ forms part of the ruler to determine hook length, but a deletion of residues 208–269 results in polyhooks with the filament attached^42^, having led to a hypothesis that residues 208–235 may contribute to efficient substrate specificity switching of the flagellar type III protein export apparatus. To verify this hypothesis, we introduced systematic deletions into FliK_L_ and found that a deletion of residues 206–235 not only shortened hook length according to the size of deletion but also caused a loose hook length control (Fig. 2). The hook length control became much worse in the *fliK*(∆206–255) and *fliK*(∆206–265) mutants (Fig. 3c). The deletion of residues 206–265 considerably reduced the export switching function of FliK_C_ (Fig 3d,e). An insertion of 100 amino-acids between Glu-270 and Trp-271 residues in the core domain of FliK_C_ considerably improved the switching function of FliK(∆2-99 + ∆206-265), thereby shortening the hook length considerably and increasing the probability of filament formation significantly (Figs. 4 and 5). Consistently, this inserted sequence allowed FliK(∆2-99 + ∆206-265 + I304pBPA) to form a photo-crosslinked product with FlhB_C_ in a way similar to FliK(I304pBPA) (Fig. 4e). Therefore, we suggest that the inserted sequence of the suppressor mutant significantly increases the binding affinity of the core domain of FliK_C_ for FlhB_C_. Although FliK(∆2-99) is not secreted via the flagellar type III protein export apparatus during hook assembly, it retains the ability to catalyze the substrate specificity switching of the flagellar type III protein export apparatus to a considerable degree^49^. Therefore, we suggest that FliK_L_ is also required for efficient interaction between the core domain of FliK_C_ and FlhB_C_. When the length of FliK_L_ was shortened by deletions, the export switching activity of FliK_C_ was reduced depending on the size of deletions (Fig. 3). Furthermore, when the linker length became longer by 40 amino-acids compared to the wild-type length, the switching function of FliK_C_ became worse (Fig. 6). Therefore, we propose that a proper length of FliK_L_ between FliK_N_ and FliK_C_ may be important for FliK_C_ to bind to FlhB_C_ to switch the substrate specificity of the flagellar type III protein export apparatus. Assuming that FliK_N_ suppresses the switching activity of FliK_C_ when FliK_N_ gets close to FliK_C_ via deletion of residues in FliK_L_, it is also possible that FliK_L_ may push FliK_N_ away from FliK_C_ to allow these two domains to fully exert their own functions.

The core domain of FliK_C_ consists of four β-strands, β1, β2, β3 and β4 and two α-helices, α1 and α2. Three parallel β1, β3 and β4 strands and one anti-parallel β2 strand form a hydrophobic core with the α1 and α2 helices (Fig. 4d)^46^. Highly conserved Val-302 and Ile-304 residues in the β2 strand, which are critical for the switching function of FliK^47^, form hydrophobic interaction networks in FliK_C_^46^. Photo-crosslinking experiments have shown that Val-302 and Ile-304 are in very close proximity to FlhB_C_, suggesting that these two residues are exposed on the molecular surface of FliK_C_ upon binding to FlhB_C_^45^. Since FliK(∆2-99 + ∆206-265SP + I304pBPA) formed a photo-crosslinked product with FlhB_C_ whereas neither FliK(∆2-99 + I304pBPA) nor FliK(∆2-99 + ∆206-265 + I304pBPA) did (Fig. 4e), this suggests that the inserted sequence of the suppressor mutant induces a conformational change of the N-terminal portion of the core domain of FliK_C_ to allow Ile-304 to bind to FlhB_C_. Therefore, we propose that FliK_L_ may be required for efficient conformational rearrangements of FliK_C_ to interact with FlhB_C_. However, it is also possible that deletion of residues 206–265 makes FliK_N_ very close to FliK_C_ to suppress the interaction of FliK_C_ with FlhB_C_ and that the inserted sequence of the suppressor mutant may push FliK_N_ away from FliK_C_, allowing FliK to bind to FlhB_C_.

FliK_C_ associates with and dissociates from FliK_N_ in solution^41^. When FliK_L_ adopts a fully extended conformation, the N-terminal portion of the core domain of FliK_C_ becomes disordered^41^. Because the length of the most extended polypeptide is 0.37 nm per residue, the stretch of FliK sequence inside the channel of the hook-basal body must be longer than 250 residues to measure the hook length of about 55 nm together with the rod length of 35 nm. Therefore, we propose that FliK_L_ may adopt a fully extended conformation when the hook length reaches about 55 nm, allowing Val-302 and Ile-304 in the hydrophobic core domain of FliK_C_ to be in very close proximity to FlhB_C_ to catalyze substrate specificity switching of the flagellar type III protein export apparatus.

## Methods

### Bacterial strains, plasmids and media

Bacterial strains and plasmids used in this study are listed in Table 1. To construct the *Salmonella fliK*(∆206–235), *fliK*(∆206–245), *fliK*(∆206–255) and *fliK*(∆206–265) mutant strains, the *fliK* gene on the chromosome was replaced by the *fliK*(∆206–235), *fliK*(∆206–245), *fliK*(∆206–255) and *fliK*(∆206–265) alleles, using the λ Red homologous recombination system^56^. L-broth contained 10 g of Bacto-Tryptone, 5 g of yeast extract and 5 g of NaCl per liter. Soft agar plates contained 10 g of Bacto Tryptone, 5 g of NaCl and 0.35% Bacto-Agar per liter. Ampicillin was added at a final concentration of 100 μg/ml if necessary.

### DNA manipulations

DNA manipulations were carried out as described^57^. A series of mutant variants of FliK with deletions within FliK_L_ were generated by inverse PCR using pKM002^48^ or pNM201^44^ as a template. The *fliK*(∆206–265SP) allele were generated by overlap PCR method. All of the *fliK* deletions were confirmed by DNA sequencing. DNA sequencing reactions were carried out using BigDye v3.1 (Applied Biosystems) and then the reaction mixtures were analyzed by a 3130 Genetic Analyzer (Applied Biosystems).

### Motility assays in soft agar

Fresh colonies were inoculated onto 0.35% soft tryptone agar plates and incubated at 30 °C. At least seven independent measurements were carried out.

### Secretion assays

Secretion assays were performed as described previously^58^. *Salmonella* cells were grown in 5 ml L-broth containing 100 μg/ml ampicillin at 30 °C with shaking until the cell density had reached an OD_600_ of ca. 1.2–1.6. 1.5 ml of each culture was transferred into a 1.5 ml Eppendorf tube. After centrifugation (15,000 g, 5 min, 4 °C), cell pellets and culture supernatants were collected, separately. The cells were resuspended in OD_600_ × 250 μl of SDS-loading buffer (62.5 mM Tris-HCl, pH 6.8, 2% SDS, 10% glycerol, 0.001% bromophenol blue) containing 1 μl of 2-mercaptoethanol and heated at 95 °C for 3 min. Trichloroacetic acid was added to each culture supernatant at a final concentration of 10%. After leaving on ice for 1 h, proteins in the culture supernatants were precipitated by centrifugation at 20,000 g for 20 min. Pellets were suspended in OD_600_ x 25 μl of a Tris-SDS loading buffer (one volume of 1 M Tris, nine volumes of 1 × SDS loading buffer) containing 1 μl of 2-mercaptoethanol and heated at 95 °C for 3 min. After sodium dodecyl sulfate-polyacrylamide gel electrophoresis (SDS-PAGE), immunoblotting with polyclonal anti-FlgE, anti-FliC or anti-FliK antibody was carried out as described previously^16^. Detection was done with an ECL prime western blotting detection reagent (GE Healthcare). Chemiluminescence signals were captured by a Luminoimage analyzer LAS-3000 (GE Healthcare). The regions of interest were cropped from original immunoblots shown in the Supplemental information using a software, Photoshop CS6, and then the contrast and brightness were adjusted. At least three independent experiments were performed.

### Electron microscopy

Osmotically shocked *Salmonella* cells were prepared described previously^45^. After centrifugation (18,500 g, 30 min), the cell pellets were resuspended in 200 μl of H_2_O. Samples were applied to carbon-coated copper grids and were negatively stained with 1.0% (W/V) phosphotungstic acid, pH 7.0. Micrographs were recorded at a magnification of × 5,000 with a JEM-1200EXII transmission electron microscope (JEOL, Tokyo, Japan) operating at 80 kV.

Hook-basal bodies and polyhook-basal bodies were isolated as described before^27^. *Salmonella* cells were grown in 5 l L-broth containing ampicillin at 30 °C with shaking until the cell density had reached an OD_600_ of ca. 1.0. The cells were harvested by centrifugation (10,000 g, 10 min, 4 °C) and suspended in 20 ml of ice-cold 0.1 M Tris-HCl pH 8.0, 0.5 M sucrose, followed by addition of EDTA and lysozyme at the final concentrations of 10 mM and 0.1 mg/ml, respectively. The cell suspensions were stirred for 30 min at 4 °C and then were solubilized on ice for 1 hour by adding Triton X-100 and MgSO_4_ at final concentrations of 1% and 10 mM, respectively. The cell lysates were adjusted to pH 10.5 with 5 M NaOH and then centrifuged (10,000 g, 20 min, 4 °C) to remove cell debris. After ultracentrifugation (45,000 g, 60 min, 4 °C), pellets were resuspended in 10 mM Tris-HCl, pH 8.0, 5 mM EDTA, 1% Triton X-100, and the solution was loaded a 20–50% (w/w) sucrose density gradient in 10 mM Tris-HCl, pH 8.0, 5 mM EDTA, 1% Triton X-100. After ultracentrifugation (49,100 g, 13 h, 4 °C), intact flagella were collected and ultracentrifuged (60,000 g, 60 min, 4 °C). Pellets were suspended in 50 mM glycine, pH 2.5, 0.1% Triton X100, and were incubated at room temperature for 30 min to depolymerize flagellar filaments. After ultracentrifugation (60,000 g, 60 min, 4 °C), pellets were resuspended in 50 μl of 10 mM Tris-HCl, pH 8.0, 5 mM EDTA, 0.1% Triton X100. Samples were negatively stained with 2%(w/v) uranyl acetate. Samples were applied to carbon-coated copper grids and were negatively stained with 2%(w/v) uranyl acetate. Electron micrographs were recorded with a JEM-1011 transmission electron microscope (JEOL, Tokyo, Japan) operated at 100 kV and equipped with a F415 CCD camera (TVIPS, Gauting, Germany). Hook length was measured by ImageJ version 1.48 (National Institutes of Health).

### Photo-crosslinking

*E. coli* BL21(DE3) cells harboring pEVOL^59^ and a pETDuet-based plasmid encoding both FliK with an amber mutation and FlhB_C_ were exponentially grown at 30 °C in L-broth containing 1 mM pBPA. Then, 100 μM IPTG and 0.02% arabinose were added and the incubation was continued until the culture density had reached an OD_600_ of ca. 1.4–1.5. Photo-crosslinking was carried out as described previously^60^. The cell pellets were harvested by centrifugation, suspended in SDS-loading buffer, and heated at 95 °C for 3 min. After SDS-PAGE, immunoblotting with polyclonal anti-FliK antibody was carried out. Detection was done with an ECL prime western blotting detection reagent. Chemiluminescence signals were captured by a Luminoimage analyzer LAS-3000. The regions of interest were cropped from original immunoblots shown in the Supplemental information using a software, Photoshop CS6, and then the contrast and brightness were adjusted. At least three independent experiments were performed.

## Supplementary information

Supplementary Figures.

## Data Availability

All data generated or analyzed during this study are included in this published article and its Supplementary Information files.
