# Supplementary material for: The flexible linker of the secreted FliK ruler is required for export switching of the flagellar protein export apparatus
Source: Sci Rep. 2020 Jan 21;10:838. doi: 10.1038/s41598-020-57782-5 (PMC6972891; doi:10.1038/s41598-020-57782-5)
Supplement: Supplementary file 1 — Supplementary Figures. [file 41598_2020_57782_MOESM1_ESM.pdf]

Supporting Information

The flexible linker of the secreted FliK ruler is required for export switching of the flagellar protein export apparatus

Miki Kinoshita, Seina Tanaka, Yumi Inoue, Keiichi Namba, Shin-Ichi Aizawa, and Tohru Minamino

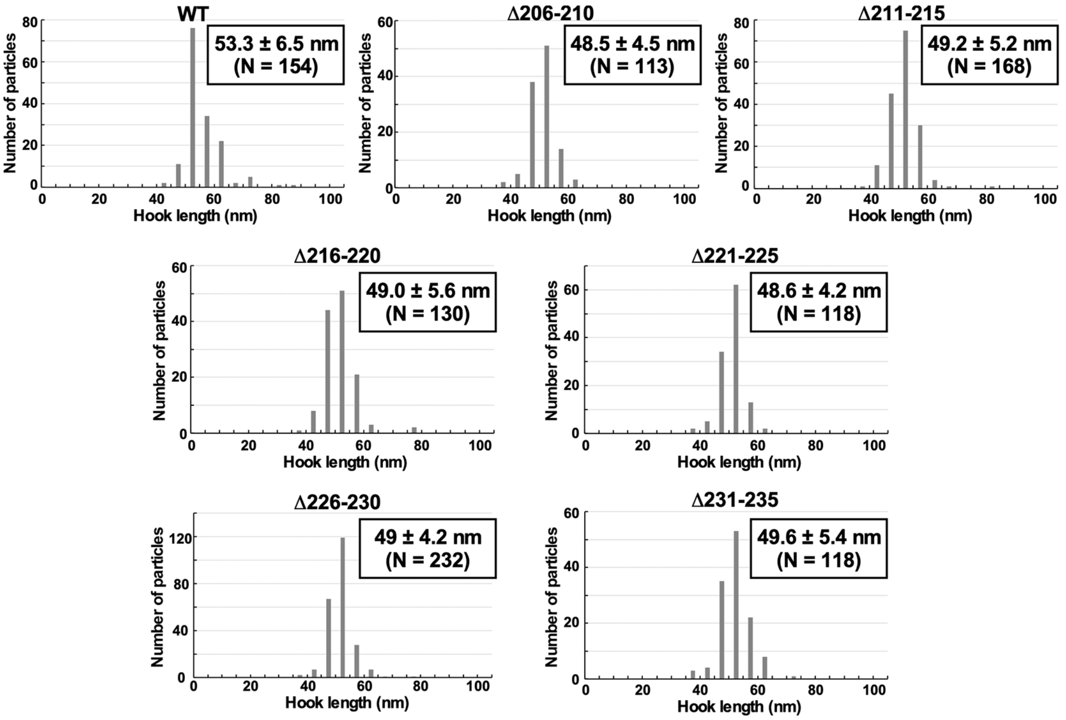

Fig. S1. Effect of in-frame deletions of five amino acids within the N-terminal region of $FliK_L$ on the hook length. Histograms of hook length distribution of TH8426 harboring pMK002 (WT), pMMK1001 (Δ206–210), pMMK1002 (Δ211–215), pMMK1003 (Δ216–220), pMMK1004 (Δ221–225), pMMK1005 (Δ226–230) or pMMK1006 (Δ231–235).

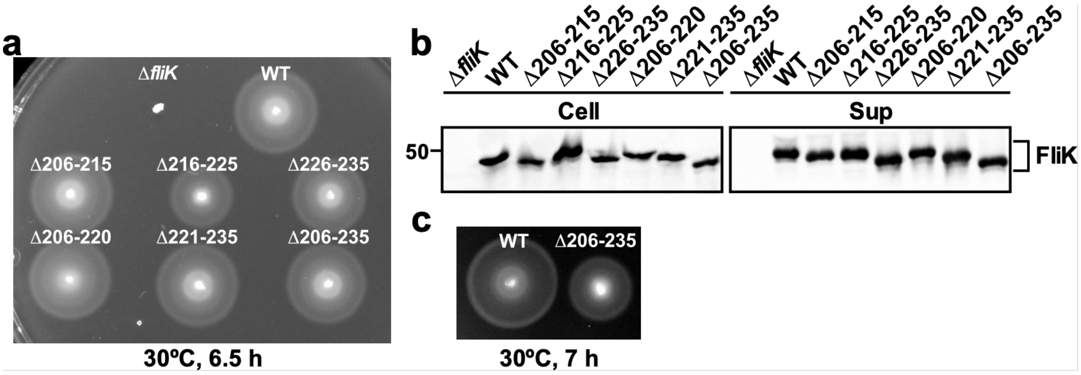

Fig. S2. Effect of in-frame deletions in $FliK_L$ on motility. (a) Motility of TH8426 harboring pTrc99AFF4 (Δ*fliK*), pMK002 (WT), pMMK1007 (Δ206–215), pMMK1008 (Δ216–225), pMMK1009 (Δ226–235), pMMK1010 (Δ206–220), pMMK1011 (Δ221–235) or pMMK1006 (Δ206–235) in soft agar. Plates were incubated at 30ºC for 6.5 hours. (b) Secretion assays of FliK. Immunoblotting using polyclonal anti-FliK antibody, of whole cell proteins (Cell) and culture supernatants (Sup) from the above strains. The position of a 50 kDa marker is shown on the left. (c) Motility of SJW1103 (WT) and MMK1012 (Δ206–235) in soft agar. Plates were incubated at 30ºC for 7 hours.

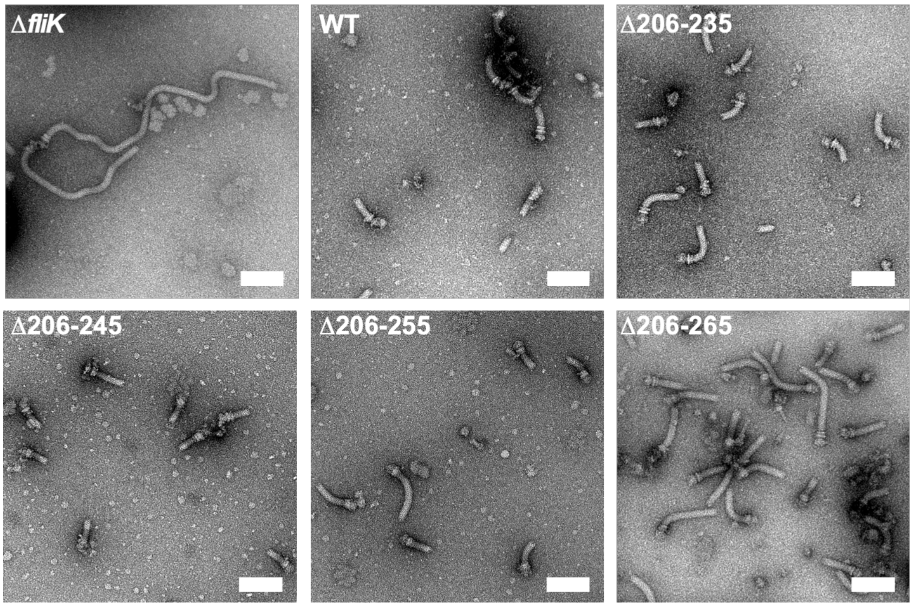

Fig. 3S. Electron micrograms of hook-basal bodies and polyhook-basal bodies isolated from TH8426 (Δ*fliK*), SJW1103 (WT), MMK1012 (Δ206–235), MMK1013 (Δ206–245), MMK1014 (Δ206–255) and MMK1015 (Δ206–265). Scale bar, 100 nm.

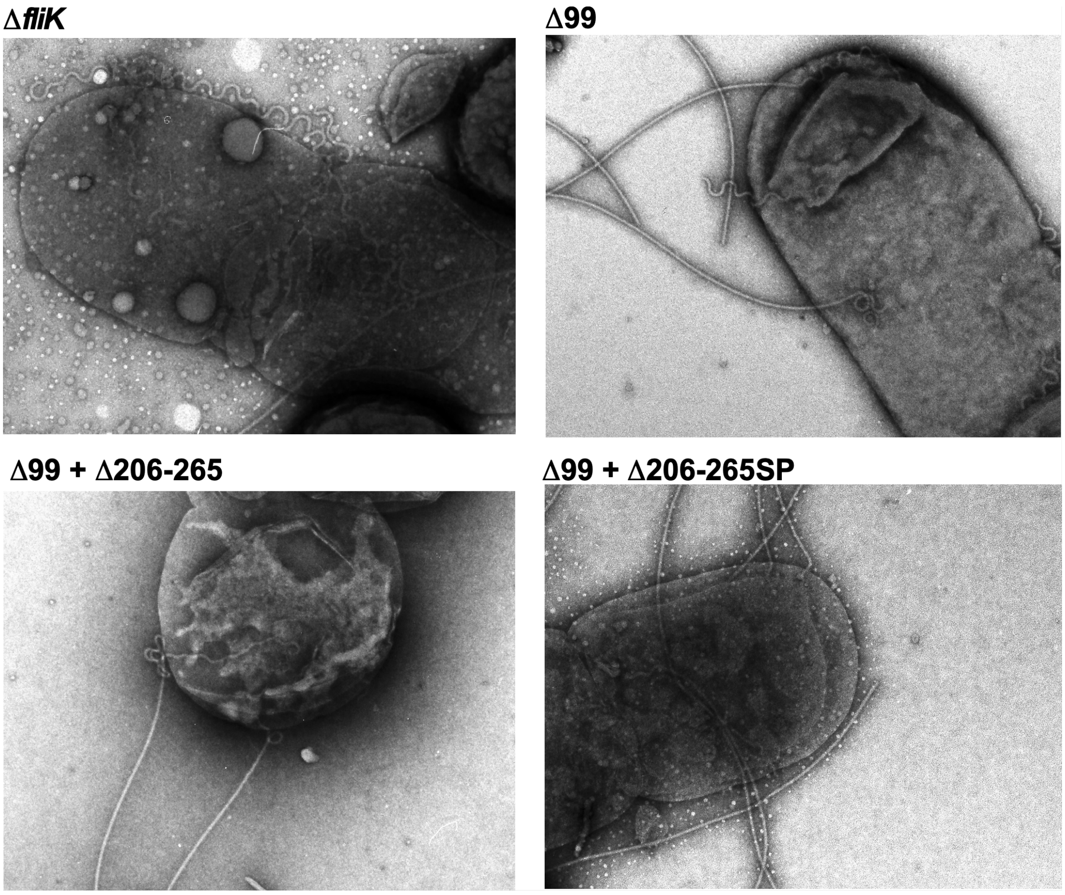

Fig. S4. Electron micrographs of TH8426 cells harboring pTrc99A (Δ*fliK*), pNM201 (Δ99), pMMK1030 (Δ99 + Δ206–265) or pMMK1030SP (Δ99 + Δ206–265SP). Cells were grown in L-broth containing ampicillin and 1mM IPTG. The cells were suspended in 500 μl of cold 50% sucrose solution. After incubation for 15 min on ice, 20 ml of H_2O was added to the cell suspensions. The cell suspensions were stirred at room temperature and then Mg^{2+} was added at a final concentration of 1 mM. After centrifugation, osmotically shocked cells were negatively stained with 1% (W/V) phosphotungstic acid (pH 7.0). Micrographs were taken at a magnification of ×5,000.

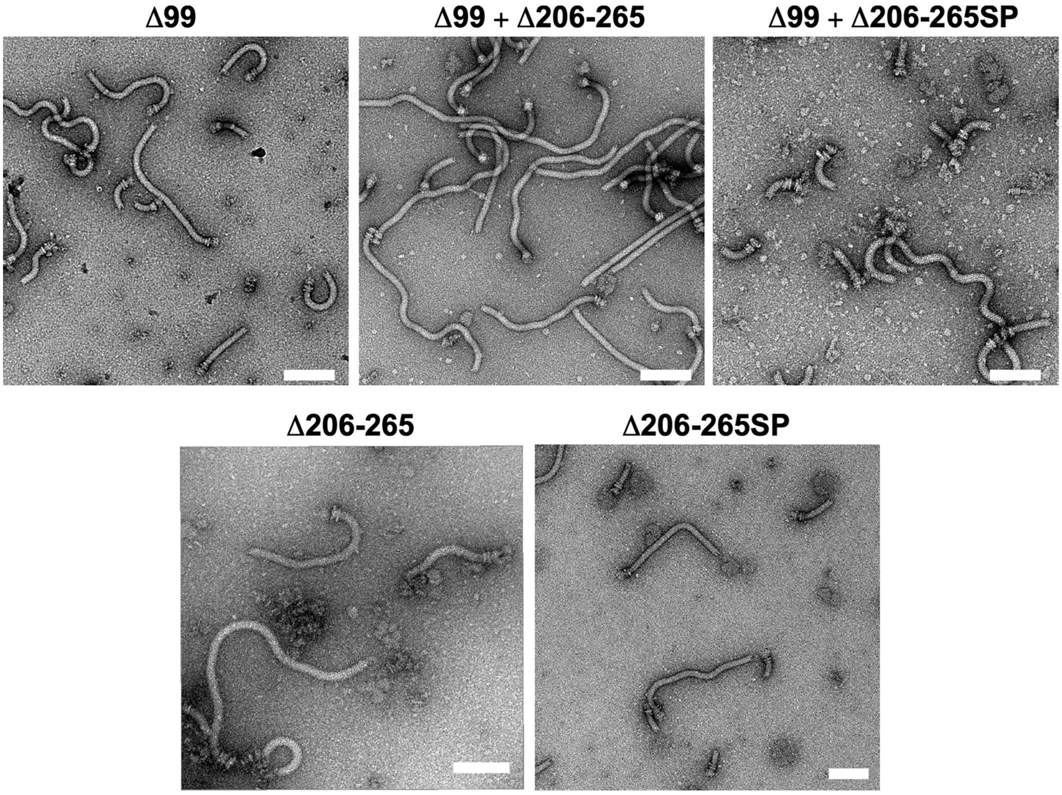

Fig. S5. Electron micrographs of hook-basal bodies and polyhook-basal bodies isolated from TH8426 harboring pNM201 (Δ99), pMMK1030 (Δ99 + Δ206–265), pMMK1030SP (Δ99 + Δ206–265SP), pMMK1015 (Δ206–265) or pMMK1015SP (Δ206–265SP). Scale bar, 100 nm.

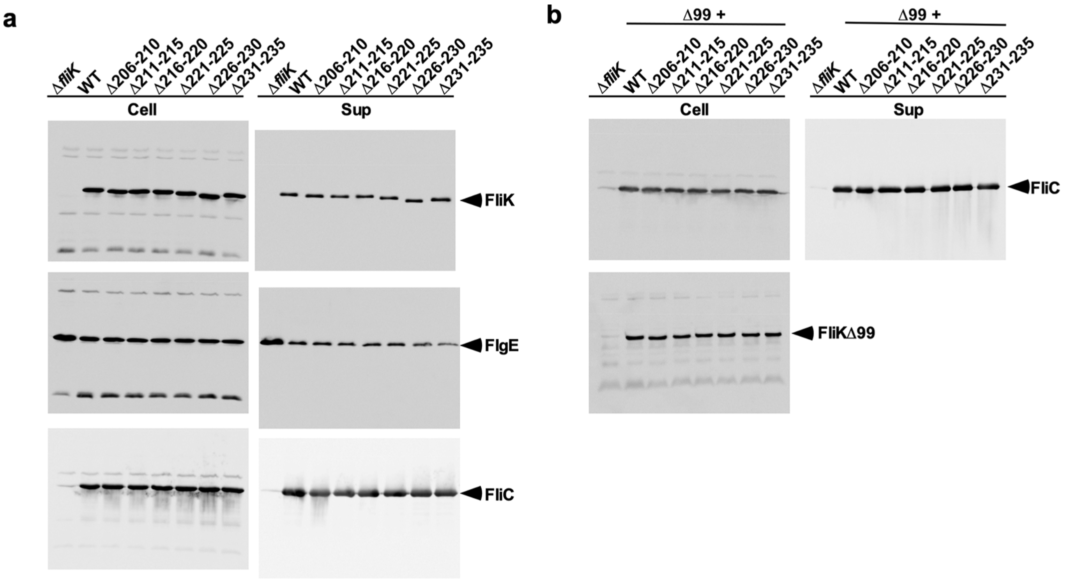

Fig. S6. Original immunoblots shown in Figure 1. (a) Effect of deletions of five residues within the N-terminal region of $FliK_L$ on FliK function. Whole cell proteins (Cell) and culture supernatant fractions (Sup) were prepared from TH8426 harboring pTrc99AFF4 (Δ*fliK*), pMK002 (WT), pMMK1001 (Δ206–210), pMMK1002 (Δ211–215), pMMK1003 (Δ216–220), pMMK1004 (Δ221–225), pMMK1005 (Δ226–230) or pMMK1006 (Δ231–235) grown exponentially at 30ºC in L-broth containing ampicillin. 8 μl of each protein sample, which was normalized to an optical density of OD_{600}, was subjected to SDS-PAGE and was analyzed by immunoblotting with polyclonal anti-FliK, anti-FlgE or anti-FliC antibody. Chemiluminescence signals were captured by a Luminoimage analyser LAS3000. (b) Effect of deletions of five residues in the N-terminal portion of $FliK_L$ on the switching function of FliK(Δ2–99). Whole cell proteins (Cell) and culture supernatants (Sup) were prepared from TH8426 transformed with pTrc99AFF4 (Δ*fliK*), pNM201 (Δ99), pMMK1016 (Δ99 + Δ206–210), pMMK1017 (Δ99 + Δ211–215), pMMK1018 (Δ99 + Δ216–220), pMMK1019 (Δ99 + Δ221–225), pMMK1020 (Δ99 + Δ226–230) or pMMK1021 (Δ99 + Δ231–235). The cells were exponentially grown in L-broth containing ampicillin and 1 mM IPTG with shaking at 30ºC. 8 μl of each protein sample, which was normalized to an optical density of OD_{600}, was subjected to SDS-PAGE and was analyzed by immunoblotting with polyclonal anti-FliC or anti-FliK antibody. Chemiluminescence signals were captured by a Luminoimage analyser LAS3000.

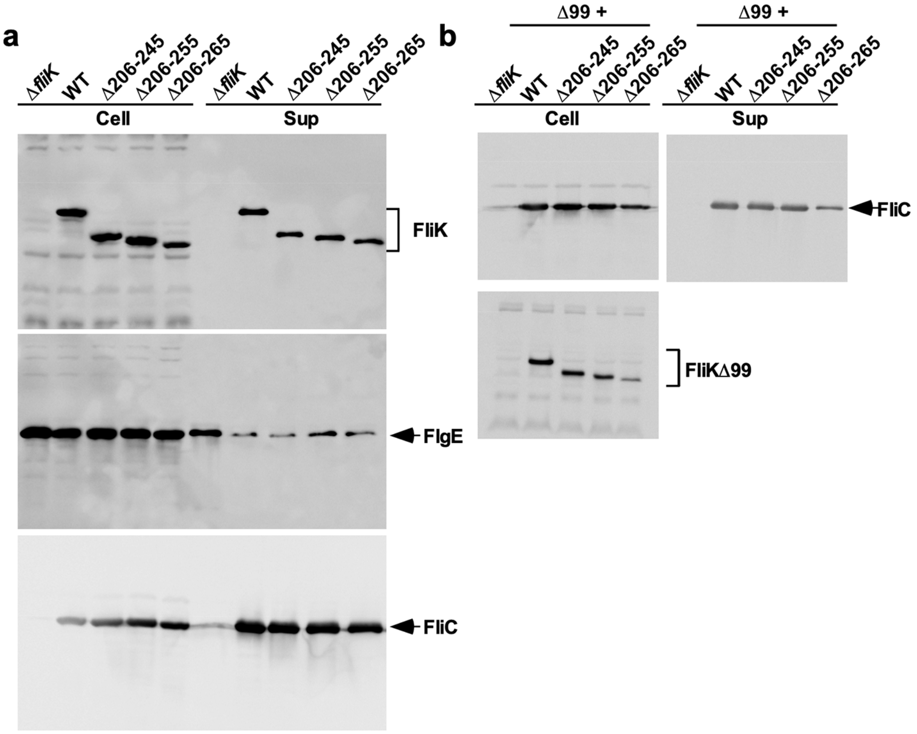

Fig. S7. Original immunoblots shown in Figure 3. (a) Effect of deletions of residues 206–245, 206–255 or 206–265 of FliK$_L$ on FliK function. Whole cell proteins (Cell) and culture supernatant fractions (Sup) were prepared from TH8426 (Δ*fliK*), SJW1103 (WT), MMK1013 (Δ206–245), MMK1014 (Δ206–255) and MMK1015 (Δ206–265) grown exponentially at 30ºC in L-broth. 8 µl of each protein sample, which was normalized to an optical density of OD$_{600}$, was subjected to SDS-PAGE and was analyzed by immunoblotting with polyclonal anti-FliK, anti-FlgE or anti-FliC antibody. Chemiluminescence signals were captured by a Luminoimage analyser LAS3000. (b) Effect of deletions of residues 206–245, 206–255 or 206–265 of FliK$_L$ on the switching function of FliK(Δ2–99). Whole cell proteins (Cell) and culture supernatants (Sup) were prepared from TH8426 harboring pTrc99AFF4 (Δ*fliK*), pNM201 (Δ99), pMMK1028 (Δ99 + Δ206–245), pMMK1029 (Δ99 + Δ206–255) or pMMK1030 (Δ99 + Δ206–265). The cells were exponentially grown in L-broth containing ampicillin and 1 mM IPTG with shaking at 30ºC. 8 µl of each protein sample, which was normalized to an optical density of OD$_{600}$, was subjected to SDS-PAGE and was analyzed by immunoblotting with polyclonal anti-FliC or anti-FliK antibody. Chemiluminescence signals were captured by a Luminoimage analyser LAS3000.

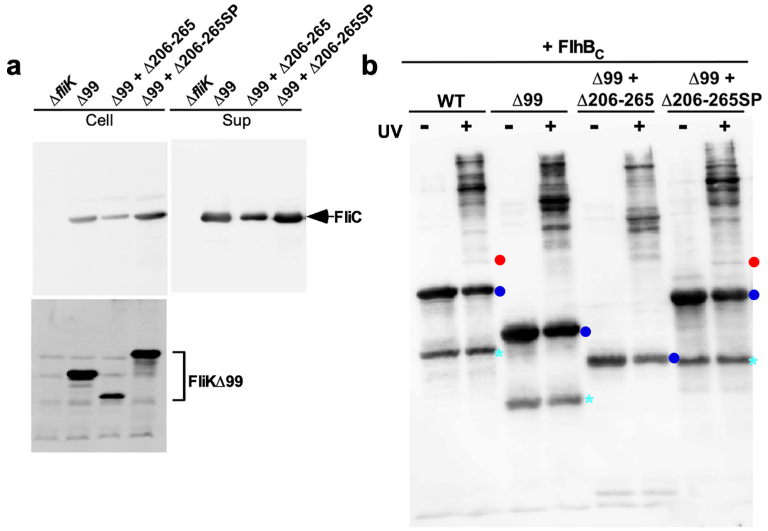

Fig. S8. Original immunoblots shown in Figure 4. (a) Secretion assay of FliC. Immunoblotting using polyclonal anti-FliC (1st row) or anti-FliK (2nd row) antibody, of whole cell proteins (Cell) and culture supernatants (Sup) prepared from TH8426 harboring pTrc99A (Δ*fliK*), pNM201 (Δ99), pMMK1030 (Δ99 + Δ206–265) or pMMK1030SP (Δ99 + Δ206–265SP). The cells were exponentially grown in L-broth containing ampicillin and 1 mM IPTG with shaking at 30ºC. 8 μl of each protein sample, which was normalized to an optical density of OD_{600}, was subjected to SDS-PAGE and was analyzed by immunoblotting with polyclonal anti-FliC or anti-FliK antibody. Chemiluminescence signals were captured by a Luminoimage analyser LAS3000. (b) Photo-crosslinking between FliK(Δ2–99) and $FlhB_C$. *E. coli* BL21(DE3) cells co-expressing FliK(I304pBPA), FliK(Δ99 + I304pBPA), FliK(Δ99 + Δ206–265 + I304pBPA) or FliK(Δ99 + Δ206–265SP + I304pBPA) with FhB_C were UV-irradiated for 5 min (+) or not irradiated (-), and then analyzed by immunoblotting with polyclonal anti-FliK antibody. The positions of free FliK and FliK-$FlhB_C$ photo-crosslinked products are shown by blue and red balls, respectively. C-terminal truncated variants of FliK are shown by cyan asterisk.

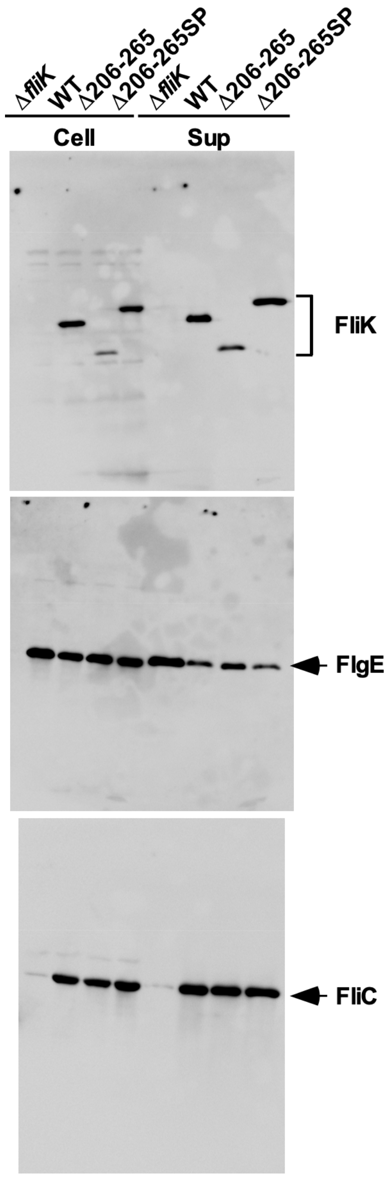

Fig. S9. Original immunoblots shown in Figure 6. Whole cell proteins (Cell) and culture supernatant fractions (Sup) were prepared from TH8426 harboring pTrc99AFF4 (Δ*fliK*), pMK002 (WT), pMMK1015 (Δ206–265) or pMMK1015SP (Δ206–265SP). 8 µl of each protein sample, which was normalized to an optical density of OD_{600}, was subjected to SDS-PAGE and was analyzed by immunoblotting with polyclonal anti-FliK, anti-FlgE or anti-FliC antibody. Chemiluminescence signals were captured by a Luminoimage analyser LAS3000.
